# Exosome-transmitted lncRNA UFC1 promotes non-small-cell lung cancer progression by EZH2-mediated epigenetic silencing of PTEN expression

**DOI:** 10.1038/s41419-020-2409-0

**Published:** 2020-04-02

**Authors:** Xueyan Zang, Jianmei Gu, Jiayin Zhang, Hui Shi, Sinan Hou, Xueying Xu, Yanke Chen, Yu Zhang, Fei Mao, Hui Qian, Taofeng Zhu, Wenrong Xu, Xu Zhang

**Affiliations:** 1 Jiangsu Key Laboratory of Medical Science and Laboratory Medicine, School of Medicine, Jiangsu University, 301 Xuefu Road, 212013 Zhenjiang, Jiangsu China; 2 Departmemt of Clinical Laboratory Medicine, Nantong Tumor Hospital, 30 Tongyang North Road, 226361 Nantong, Jiangsu China; 3 Department of Respiratory Medicine, the Affiliated Yixing Hospital of Jiangsu University, 75 Tongzhenguan Road, 214200 Yixing, Jiangsu China

**Keywords:** Non-small-cell lung cancer, Diagnostic markers

## Abstract

Long non-coding RNAs (LncRNAs) have been suggested as important regulators of cancer development and progression in non-small cell lung cancer (NSCLC). Nevertheless, the biological roles and clinical significance of lncRNA UFC1 in NSCLC remain unclear. We detected the expression of UFC1 in tumor tissues, serum, and serum exosomes of NSCLC patients by qRT-PCR. Gene overexpression or silencing were used to examine the biological roles of UFC1 in NSCLC. RNA immunoprecipitation and ChIP assays were performed to evaluate the interaction between UFC1 and enhancer of zeste homolog 2 (EZH2) and the binding of EZH2 to PTEN gene promoter. Rescue study was used to access the importance of PTEN regulation by UFC1 in NSCLC progression. UFC1 expression was upregulated in tumor tissues, serum, and serum exosomes of NSCLC patients and high level of UFC1 was associated with tumor infiltration. UFC1 knockdown inhibited NSCLC cell proliferation, migration and invasion while promoted cell cycle arrest and apoptosis. UFC1 overexpression led to the opposite effects. Mechanistically, UFC1 bound to EZH2 and mediated its accumulation at the promoter region of PTEN gene, resulting in the trimethylation of H3K27 and the inhibition of PTEN expression. UFC1 knockdown inhibited NSCLC growth in mouse xenograft tumor models while the simultaneous depletion of PTEN reversed this effect. NSCLC cells derived exosomes could promote NSCLC cell proliferation, migration and invasion through the transfer of UFC1. Moreover, Exosome-transmitted UFC1 promotes NSCLC progression by inhibiting PTEN expression via EZH2-mediated epigenetic silencing. Exosome-mediated transmit of UFC1 may represent a new mechanism for NSCLC progression and provide a potential marker for NSCLC diagnosis.

## Introduction

Lung cancer is the first cause of malignant tumor-related death, accounting for ~20% of tumor death cases^1^. Non-small cell lung cancer (NSCLC), including adenocarcinoma, squamous cell carcinoma, and large cell carcinoma, accounts for ~80–85% of all lung cancer cases. Despite the advances in diagnostic and therapeutic strategies, the prognosis of lung cancer remains poor and the survival of lung cancer patients is not satisfactory. Therefore, it is of great significance to understand the detailed molecular mechanism for the pathogenesis of NSCLC and to identify new markers for NSCLC diagnosis and therapy to reduce the morbidity and mortality of this devastating disease.

Long non-coding RNAs (LncRNAs) are defined as a class of ncRNAs with more than 200 nucleotide length. LncRNAs participate in multiple cellular processes such as cell cycle progression, apoptosis, genome stability^2–5^. Accumulating studies have shown that the dysregulation of lncRNAs is involved in cancer development, progression, metastasis and drug resistance^6–9^. Fox example, LINC00968 acts as an oncogene in NSCLC by activating Wnt signaling pathway^10^. DANCR acts as a ceRNA for miR-496 to regulate the expression of mTOR, thus promoting NSCLC growth^11^. LINC00473 is highly induced by LKB1 inactivation and LINC00473 upregulation promotes the growth of LKB1-inactivated NSCLC cells^12^. These findings suggest that lncRNAs are key players in the pathogenesis of NSCLC and potential novel biomarkers for NSCLC diagnosis and therapy.

LncRNA UFC1 was initially found to be increased in hepatocellular carcinoma (HCC) tissues and could upregulate β-catenin protein translation by binding to HuR protein^13^. Yu et al. reported that UFC1 knockdown suppressed proliferation and induced apoptosis of colorectal cancer (CRC) cells through the inhibition of β-catenin and the activation of p38 signaling pathways^14^. In cervical cancer, UFC1 upregulated FOXP3 expression through competitively binding miR-34a, promoting cervical cancer growth and metastasis^15^. We reported that UFC1 was upregulated in gastric cancer and UFC1 promoted GC progression by acting as a miR-498 sponge to activate Lin28b expression^16^. However, the expression and role of UFC1 in NSCLC have not been characterized. In this study, we found that UFC1 expression level was increased in the tumor tissues, serum and serum exosomes of NSCLC patients. High level of UFC1 was associated with tumor infiltration. More importantly, we found that exosome-transmitted UFC1 could bind to EZH2 to downregulate PTEN gene expression and activate PI3K/Akt signaling pathway, consequently promoting the tumorigenesis of NSCLC.

## Materials and methods

### Patients and tissue samples

A total of 66 paired tumor tissues and adjacent non-tumor tissues were collected from NSCLC patients who underwent surgery at Nantong Tumor Hospital from June 2015 to August 2017. Blood samples were collected from 40 healthy donors, 42 pneumonia patients, and 54 NSCLC patients. The use of clinical samples was approved by the ethics committee of Jiangsu University (ref no.2017003) and informed consent was obtained from all patients. All the experiments were performed in accordance with The Code of Ethics of the World Medical Association (Declaration of Helsinki). All the collected tissue samples were immediately snap-frozen in liquid nitrogen and stored at −80 °C for future use. Blood samples were centrifuged at 3000 rpm for 15 min and the serum samples were separated and kept at −20 °C.

### Cell culture

Human NSCLC cell lines (A549 and H1299) and human normal embryonic lung fibroblast (MRC-5) were purchased from the Cell Bank of the Chinese Academy of Sciences (Shanghai, China) and Gefan Biological Technology (Shanghai, China). A549 and MRC-5 cells were cultured in high glucose-DMEM with 10% fetal bovine serum (FBS; Invitrogen, Carlsbad, CA, USA). H1299 cells were cultured in RPMI 1640 medium (Invitrogen) containing 10% FBS. All the cells were cultured in a 37 °C incubator with 5% CO_2_ atmosphere.

### RNA extraction and quantitative real-time polymerase chain reaction

Total RNA was extracted from tissues or cultured cells using TRIzol reagent (Invitrogen). Total RNA in serum was purified using miRNeasy Serum/Plasma kit according to the manufacturer’s instructions (Qiagen). Exosomal RNA was isolated from serum using miRNeasy Micro Kit (Qiagen). Purity and concentration of total RNA was evaluated using NanoDrop One spectrophotometer (Thermo). RNA was reversely transcribed to cDNA by using HiScript 1st Strand cDNA Synthesis Kit (Thermo). Exosomal RNA was reversely transcribed with miScript II RT Kit (QIAGEN). Quantitative real-time polymerase chain reaction (qRT-PCR) was conducted with UltraSYBR Mixture (Cwbio, Beijing, China) on a Real-time PCR Detection System (CFX96, Bio-Rad). U6 was used as internal control. The results were expressed as threshold cycle (Ct) values and then converted to fold changes. The sequences of primers were provided in Additional file 1: Table S1.

### Exosomes isolation and detection

Exosomes were isolated from serum samples and NSCLC cells following our previous protocol^16^. The size and concentration of exosomes were determined using Nanoparticle tracking analysis (NTA) (Nanosight LM10).The morphology of isolated exosomes was identified by transmission electron microscopy (Tecnai 12; Philips).

### Cell transfection

A549 and H1299 cells were seeded into 6-well plates (2 × 10^5^/well) and cultured in 37 °C incubator overnight. SiRNAs and shRNAs were transfected into NSCLC cells using LipoFiter transfection reagent (Hanbio) in serum-free medium. At 6 h after transfection, cells were changed to complete medium and then cultured for another 30 h.The sequences of shRNAs and siRNAs were shown in Additional file 1: Table S2.

### Cell counting and colony formation assays

The transfected cells were seeded in 24-well plates (1 × 10^4^/well) and counted every 24 h for 6 days. The results are expressed as the mean values of three independent experiments. For colony formation assay, the transfected cells were cultured in six-well plates (1 × 10^3^/well) and the medium was changed every 2 days. After 10 days, the colonies were fixed with 4% paraformaldehyde and stained with crystal violet. The number of colonies was calculated under a microscope.

### Cell cycle and cell apoptosis analysis

Cell cycle analysis was performed at 36 h after transfection with a cell cycle detection kit (Fcmacs, Jiangsu, China). The collected cells were fixed in 95% ethanol overnight and then stained with 50 μg/ml propidium iodide (PI) for 30 min in dark. Flow cytometry was used to count the percentage of the cells in different phases. For cell apoptosis analysis, the transfected cells were digested, stained with an Annexin V-Alexa Fluor 647/PI apoptosis detection kit (Fcmacs, Jiangsu, China). The cell apoptosis rate was analyzed by flow cytometry.

### Transwell migration and matrigel invasion assays

The transfected cells were harvested and resuspended in serum-free medium. Cells (2 × 10^4^ for migration assay and 1 × 10^4^ for invasion assay) were plated into the upper chamber of an insert. The lower chamber was added with medium containing 10% FBS. For cell invasion assay, the upper chambers of transwell were pre-coated with diluted matrigel (BD Biosciences, New Jersey, USA). After incubation for 24 h, the migrated and invaded cells were fixed with 4% paraformaldehyde and stained with crystal violet, counted and photographed. Each well was counted with five random fields. The experiments were repeated three times.

### Western blot

Cells were lysed using RIPA buffer (Beyotime, Shanghai, China) containing protease inhibitors (Roche, CA, USA). Equal amounts of proteins were separated by 12% sodium dodecyl sulfate-polyacrylamide gel electrophoresis (SDS-PAGE), then transferred to 0.22μm PVDF membranes (Millipore), and blocked with 5% non-fat milk. The membranes were incubated with primary antibodies against N-cadherin, E-cadherin, slug, cyclin D1, twist, Bcl-2, snail (Cell Signaling Technology, Shanghai, China) at 4 °C overnight, followed by incubation with goat anti-rabbit secondary antibody for 2 h at room temperature. GAPDH was used as the loading control. Signal detection was carried out with chemiluminescence (Millipore, Billerica, MA, USA).

### RNA immunoprecipitation

RNA immunoprecipitation (RIP) was performed with a MagnaRIP kit (Millipore) following the manufacturer’s instruction. The supernatants of cell extracts were pre-cleared and then incubated with the beads for 6 h. The beads were washed with the RIP wash buffer for six times and incubated with EZH2 antibody or IgG (Millipore).The immunoprecipitated RNA were used for qRT-PCR analysis.

### Chromatin immunoprecipitation assays

Chromatin immunoprecipitation (ChIP) assay was conducted using the EZ-ChIP kit (Millipore). Cells were treated with formaldehyde and incubated for 10 min to generate DNA-protein crosslinks. Then the cell lysates were sonicated to generate chromatin fragments of 200–300 bp and immunoprecipitated with EZH2 or H3K27me3-specific antibodies (Millipore) or IgG as control. The precipitated chromatin DNA were analyzed by qRT-PCR assays. The information of the sequences of ChIP primers are listed in Additional file 1: Table S3.

### In vivo tumor formation assay

A total of 24 male BALB/c nude mice of 4 weeks (weight, 21–25 g) old were purchased from Nanjing Model Animal Center (Nanjing, China) and maintained in specific pathogen-free (SPF) conditions in accordance with the NIH guide for the care and the use of mice. The mice received sterile rodent chow and water ad libitum and were housed in sterile filter top cages with 12 h light/dark cycles. All the mice were divided randomly into three groups (eight mice/group). The mice were subcutaneously injected with A549 cells transfected with sh-UFC1 or sh-control, or co-transfected with sh-UFC1 plus si-PTEN (sh-UFC1 + si-PTEN) (2 × 10^6^ cells/mice). Tumor sizes and volumes were monitored every three days. The tumor volume was calculated using the following formula: *V*(cm^3^) = 1/2 × length × width^2^. The protocol for the animal study was approved by the Laboratory Animal Management Committee of Jiangsu University and also meet the guidelines of the National Institutes of Health Guide for the Care and Use of Laboratory Animals (UJS-LAER-2017042301). No tumor formation was observed in one mouse of sh-UFC1 group.

### Statistical analysis

SPSS 22.0 software (Chicago, IL, USA) and GraphPad Prism 5.0 (La Jolla, CA, USA) were used to analyze the data. The significant differences between different groups were analyzed by *t*-test or one-way ANOVA. The relationship between UFC1 and the clinicopathological features were compared by the Pearson *χ*2 test. Receiver operating characteristic (ROC) curve was used to evaluate the diagnostic value of UFC1. *P* values < 0.05 were considered as statistically significant.

## Results

### UFC1 expression is upregulated in NSCLC tissues, serum and serum exosomes

**Fig. 1:**
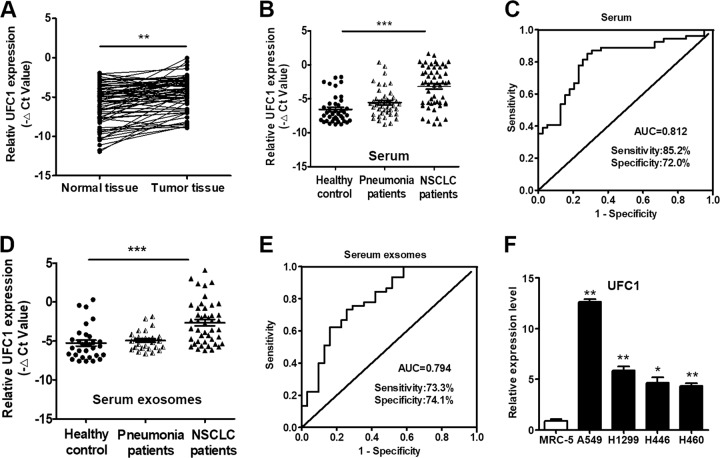
UFC1 is upregulated in the tumor tissues and serum of NSCLC patients and NSCLC cell lines. **a** UFC1 expression levels in tumor tissues and adjacent normal tissues were detected by qRT-PCR (*n* = 66). **b** UFC1 expression levels in serum of NSCLC patients, pneumonia patients, and healthy controls were detected by qRT-PCR. **c** ROC curves for the diagnostic value of serum UFC1 in NSCLC. **d** UFC1 expression levels in serum exosomes of NSCLC patients, pneumonia patients, and healthy controls. **e** ROC curves for the diagnostic value of serum exosomal UFC1 in NSCLC. **f** UFC1 expression levels in NSCLC cell lines (A549, H1299, H446, and H460) and normal embryonic lung fibroblast cells (MRC-5). *P < 0.05, **P < 0.01, ***P < 0.001.

We collected 66 pairs of human lung cancer tissues and their adjacent normal tissues to evaluate the expression levels of UFC1 by qRT-PCR. The results showed that UFC1 expression was significantly upregulated (*P* < 0.01) in NSCLC tissues compared to adjacent normal tissues (Fig. 1a). Increased UFC1 expression levels in NSCLC tissues were positively correlated with age (*P* = 0.03) and tumor infiltration (*P* = 0.02) (Additional file 1: Table S4). We then tested the expression level of UFC1 in serum samples. UFC1 expression was also upregulated in the serum of NSCLC patients compared to that of pneumonia patients and healthy donors (Fig. 1b). The receiver operating characteristic (ROC) curve was used to investigate the diagnostic value of UFC1 in serum as a biomarker for NSCLC. As shown in Fig. 1c, the area under the ROC curve (AUC) was 0.812, the sensitivity and specificity were 85.2% and 72.0%, respectively. We also detected the expression levels of UFC1 in exosomes isolated from the serum samples. The expression level of exosomal UFC1 was also increased in NSCLC patients compared to pneumonia patients and healthy donors (Fig. 1d). The area under the ROC curve (AUC) was 0.794, the sensitivity and specificity were 73.3% and 74.1%, respectively (Fig. 1e). Furthermore, we assessed the expression levels of UFC1 in NSCLC cell lines (A549, H1299, H446, and H460) and normal human embryonic lung fibroblast cell line (MRC-5). The results showed that UFC1 expression was higher in NSCLC cells than that in MRC-5 cells (Fig. 1f). Taken together, these results suggest that UFC1 expression is upregulated in NSCLC.

### UFC1 knockdown inhibits proliferation, migration, and invasion of NSCLC cells

**Fig. 2:**
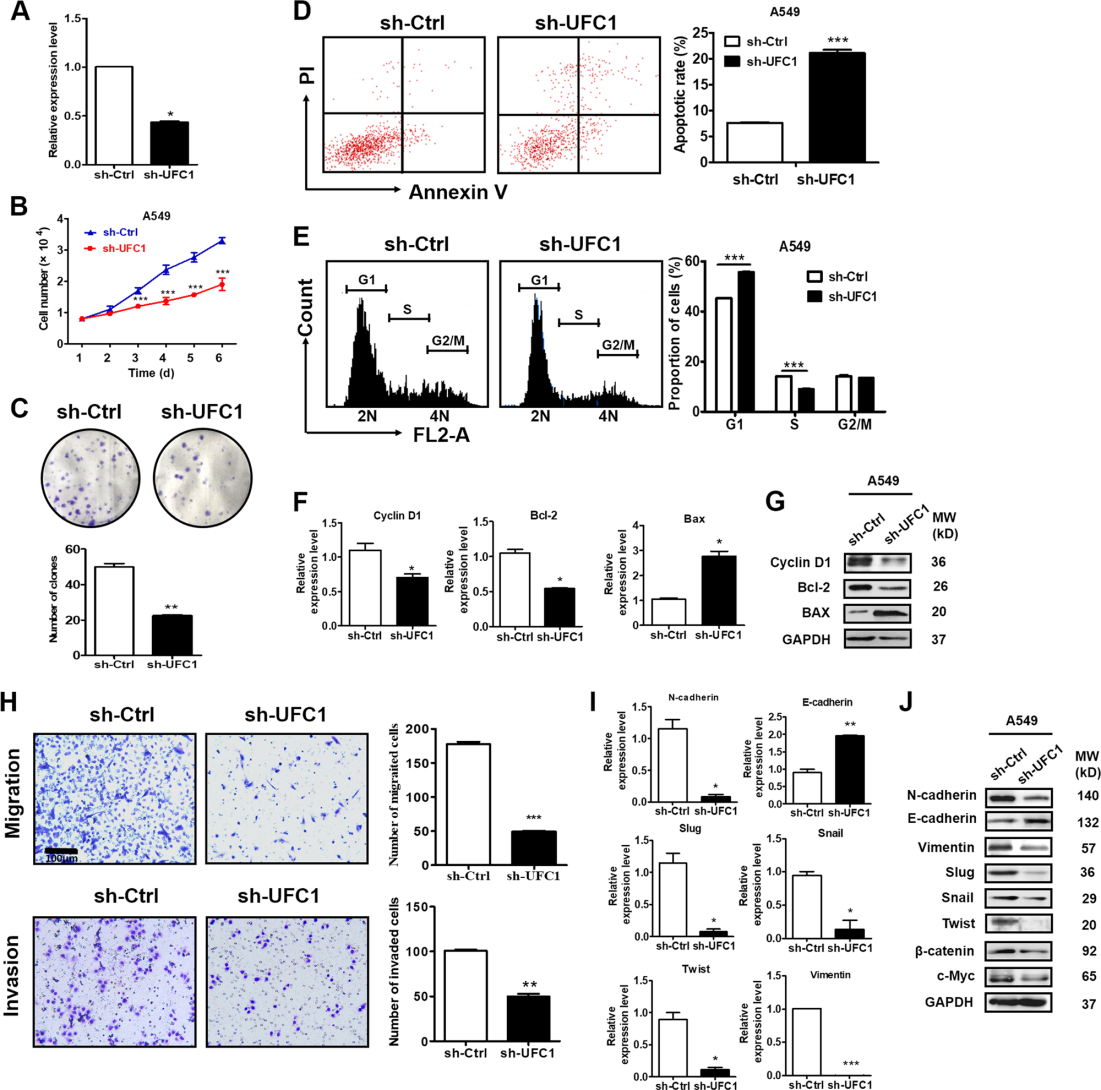
UFC1 knockdown inhibits proliferation, migration and invasion of NSCLC cells. **a** QRT-PCR analysis of UFC1 knockdown efficiency in A549 cells. **b** Cell counting assays for sh-Ctrl and sh-UFC1 transfected A549 cells. **c** Colony formation assays for sh-Ctrl and sh-UFC1 transfected A549 cells. **d** The percentage of apoptotic cells in sh-Ctrl and sh-UFC1 groups was detected by flow cytometry after Annexin V/PI staining. **e** Flow cytometric analysis of cell cycle distribution in sh-Ctrl and sh-UFC1 transfected A549 cells. **f**, **g** QRT-PCR (**f**) and western blot (**g**) analysis of cyclin D1, Bcl-2 and Bax expression in sh-Ctrl and sh-UFC1 transfected A549 cells. **h** Transwell migration and matrigel invasion assays for sh-Ctrl and sh-UFC1 transfected A549 cells. **i** QRT-PCR analysis of E-cadherin, N-cadherin, vimentin, slug, snail, and twist expression in sh-Ctrl and sh-UFC1 transfected A549 cells. **j** Western blot analysis of E-cadherin, N-cadherin, vimentin, twist, slug, snail, β-catenin, and c-Myc expression in sh-Ctrl and sh-UFC1 transfected A549 cells. The experiments were repeated for three times. Scale bar: 100 μm. *P < 0.05, **P < 0.01, ***P < 0.001.

We next wanted to know the biological roles of UFC1 in NSCLC cells. We found that UFC1 knockdown retarded the growth of A549 cells (Fig. 2a, b). The results of colony formation assay showed that the number of colonies was markedly decreased in sh-UFC1 group compared to sh-Ctrl group (Fig. 2c). We used flow cytometry to analyze cell apoptosis and cell cycle progression. The data showed that UFC1 knockdown induced an increase in the percentage of apoptotic cells (Fig. 2d) and caused a dramatic decrease in S-phase and accumulation in G1 phase of A549 cells (Fig. 2e). The results of qRT-PCR and western blot showed that the mRNA and protein levels of cyclin D1 and Bcl-2 were significantly decreased while that of Bax were increased in sh-UFC1 transfected NSCLC cells (Fig. 2f, g), indicating that UFC1 is involved in the regulation of cell apoptosis and cell cycle progression in NSCLC cells.

We further explored the effects of UFC1 on NSCLC cell migration and invasion. As shown in Fig. 2h, the number of migrated and invaded cells were decreased in sh-UFC1 group compared to sh-Ctrl group. We then detected the expression of epithelial-mesenchymal transition (EMT) related genes and proteins in A549 cells after UFC1 knockdown. The results of qRT-PCR and western blot showed that the mRNA and protein levels of E-cadherin were increased while that of N-cadherin, vimentin, slug, twist, snail were decreased in sh-UFC1 transfected group compared to sh-Ctrl group (Fig. 2i, j). In consistent with that previously reported in HCC, UFC1 knockdown also inhibited the expression of β-catenin and c-Myc in A549 cells (Fig. 2j). These results imply that UFC1 knockdown impairs the migratory and invasive abilities of NSCLC cells by suppressing EMT. The inhibitory roles of UFC1 knockdown in NSCLC cell proliferation, migration and invasion were confirmed by another set of siRNA for UFC1 (additional file 1: Fig. S1).

### UFC1 overexpression promotes NSCLC cell proliferation, migration and invasion

**Fig. 3:**
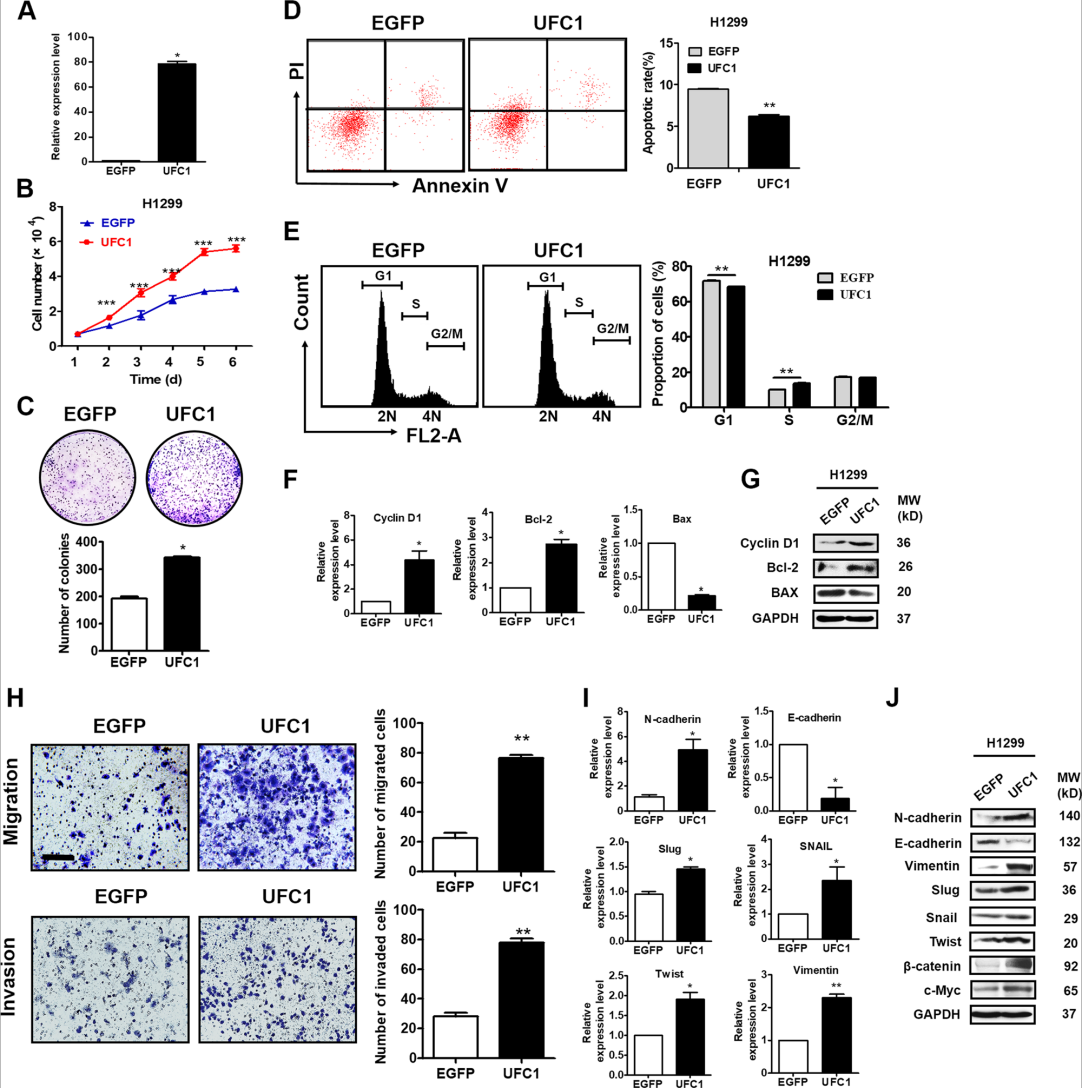
UFC1 overexpression promotes NSCLC cell proliferation, migration and invasion. **a** QRT-PCR analysis of UFC1 overexpression efficiency in H1299 cells. **b** Cell counting assays for control and UFC1 overexpressing A549 cells. **c** Colony formation assays for control and UFC1 overexpressing A549 cells. **d**, **e** Flow cytometric analysis of cell apoptosis (**d**) and cell cycle distribution (**e**) in control and UFC1 overexpressing A549 cells. **f**, **g** QRT-PCR (**f**) and western blot (**g**) analysis of cyclin D1, Bcl-2 and Bax expression in control and UFC1 overexpressing A549 cells. **h** Transwell migration and matrigel invasion assays for control and UFC1 overexpressing A549 cells. **i** QRT-PCR analysis of E-cadherin, N-cadherin, vimentin, snail, slug, and twist expression in control and UFC1 overexpressing A549 cells. **j** Western blot analysis of E-cadherin, N-cadherin, vimentin, twist, slug, snail, β-catenin, and c-Myc expression in control and UFC1 overexpressing A549 cells.The experiments were repeated for three times. Scale bar: 100 μm. *P < 0.05, **P < 0.01, ***P < 0.001.

We further performed UFC1 overexpression studies to assess the biological role of UFC1 in NSCLC (Fig. 3a). The results of cell counting and colony formation assays showed that UFC1 overexpression remarkably promoted the growth of H1299 cells (Fig. 3b, c). We also observed a significant increase in S phase and decrease of apoptosis in UFC1 overexpressing H1299 cells compared to control cells (Fig. 3d, e). The results of qRT-PCR and western blot showed that the mRNA and protein levels of cyclin D1 and Bcl-2 were significantly increased while that of Bax were decreased in UFC1 overexpressing H1299 cells (Fig. 3f, g). Furthermore, the results of migration and invasion assays showed that the number of migrated and invaded cells increased in UFC1 overexpressing H1299 cells compared to control cells (Fig. 3h). The expression of E-cadherin decreased while that of N-cadherin, vimentin, slug, snail and twist increased in H1299 cell with UFC1 overexpression (Fig. 3i, j). These data suggest that UFC1 overexpression promotes NSCLC cell proliferation, migration and invasion.

### UFC1 interacts with EZH2 to suppress PTEN expression and activate Akt pathway

**Fig. 4:**
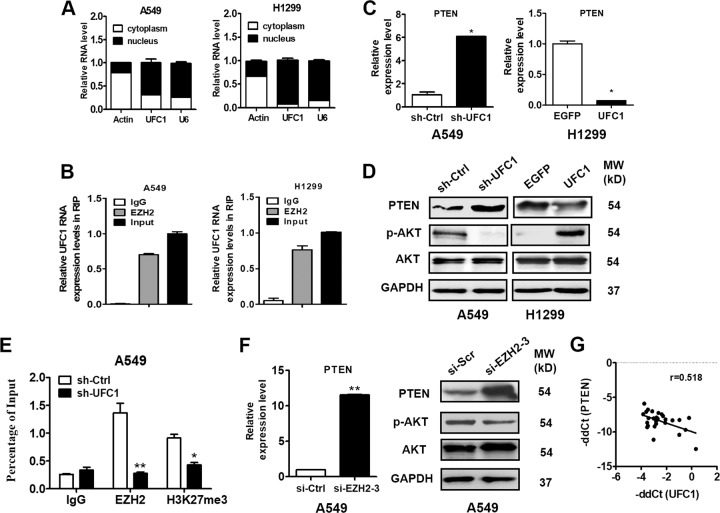
UFC1 interacts with EZH2 to suppress PTEN expression and activate Akt pathway. **a** QRT-PCR analysis of UFC1 expression levels in different subcellular fractions of A549 cells. **b** RIP assays with antibodies against EZH2 and IgG control and A549 cell extracts. RNA levels in the immunoprecipitates were detected by qRT-PCR. Expression levels of UFC1 are presented as fold enrichment to input. **c** QRT-PCR analysis of PTEN expression in NSCLC cells with UFC1 knockdown or overexpression. **d** Western blot analysis of PTEN and p-Akt expression in NSCLC cells with UFC1 knockdown or overexpression. **e** ChIP-PCR analysis of EZH2 occupancy and H3K27me3 binding in the PTEN promoter in A549 cells transfected with sh-UFC1 and sh-Ctrl. **f** QRT-PCR and western blot analysis of PTEN expression in A549 cells with EZH2 knockdown. **g** Correlation analysis of UFC1 and PTEN expression levels in tumor tissues of NSCLC patients. The experiments were repeated for three times. *P < 0.05, **P < 0.01, ***P < 0.001.

To investigate the potential mechanism for UFC1 in NSCLC, we first analyzed the distribution of UFC1 in A549 and H1299 cells and found that UFC1 expression was mainly located in the nucleus (Fig. 4a), implying that UFC1 may be involved in transcriptional regulation. To explore whether UFC1 was physically associated with EZH2 that regulated target genes at transcriptional level in the nucleus, we performed a RNA immunoprecipitation (RIP) assay using specific antibodies against EZH2 and lysates from A549 and H1299 cells. The results showed that UFC1 could be precipitated by EZH2 in A549 and H1299 cells (Fig. 4b), suggesting an interaction between UFC1 and EZH2. In consistent with the effects observed for UFC1 knockdown, the depletion of EZH2 also significantly inhibited the proliferation, migration and invasion of A549 cells (Additional file 1: Fig. S2), suggesting that EZH2 may mediate the oncogenic role of UFC1 in NSCLC cells.

EZH2 targets various tumor suppressor genes to promote cancer progression, including PTEN. We then detected the changes of PTEN expression in UFC1 knockdown or overexpressing NSCLC cells. The results of qRT-PCR showed that PTEN expression was increased in UFC1 knockdown A549 cells but decreased in UFC1 overexpressing H1299 cells (Fig. 4c). Consistently, the results of western blot showed the similar trends (Fig. 4d), implicating that PTEN inhibition may be involved in the contributions of UFC1 to NSCLC progression. Moreover, UFC1 overexpression promoted while UFC1 knockdown inhibited Akt phosphorylation in NSCLC cells (Fig. 4d). However, no change in total Akt protein level was found. We preformed chromatin immunoprecipitation assays to determine whether UFC1 suppressed the expression of PTEN by EZH2-meidated epigenetic silencing. The results showed that UFC1 knockdown suppressed EZH2 binding to the promoter of PTEN gene and EZH2-mediated histone H3 lysine 27 trimethylation (H3K27me3) modification (Fig. 4e). The mRNA and protein levels of PTEN also exhibited significant decrease in EZH2 knockdown A549 cells (Fig. 4f). Moreover, correlation analysis using 27 paired NSCLC tissues and adjacent normal tissues showed that there was a negative association between the expression of UFC1 and that of PTEN in tumor tissues (*r* = −0.518, *P* < 0.05, Fig. 4g). These results suggest that UFC1 regulates PTEN expression by binding to EZH2.

### UFC1 promotes NSCLC progression via the regulation of PTEN

**Fig. 5:**
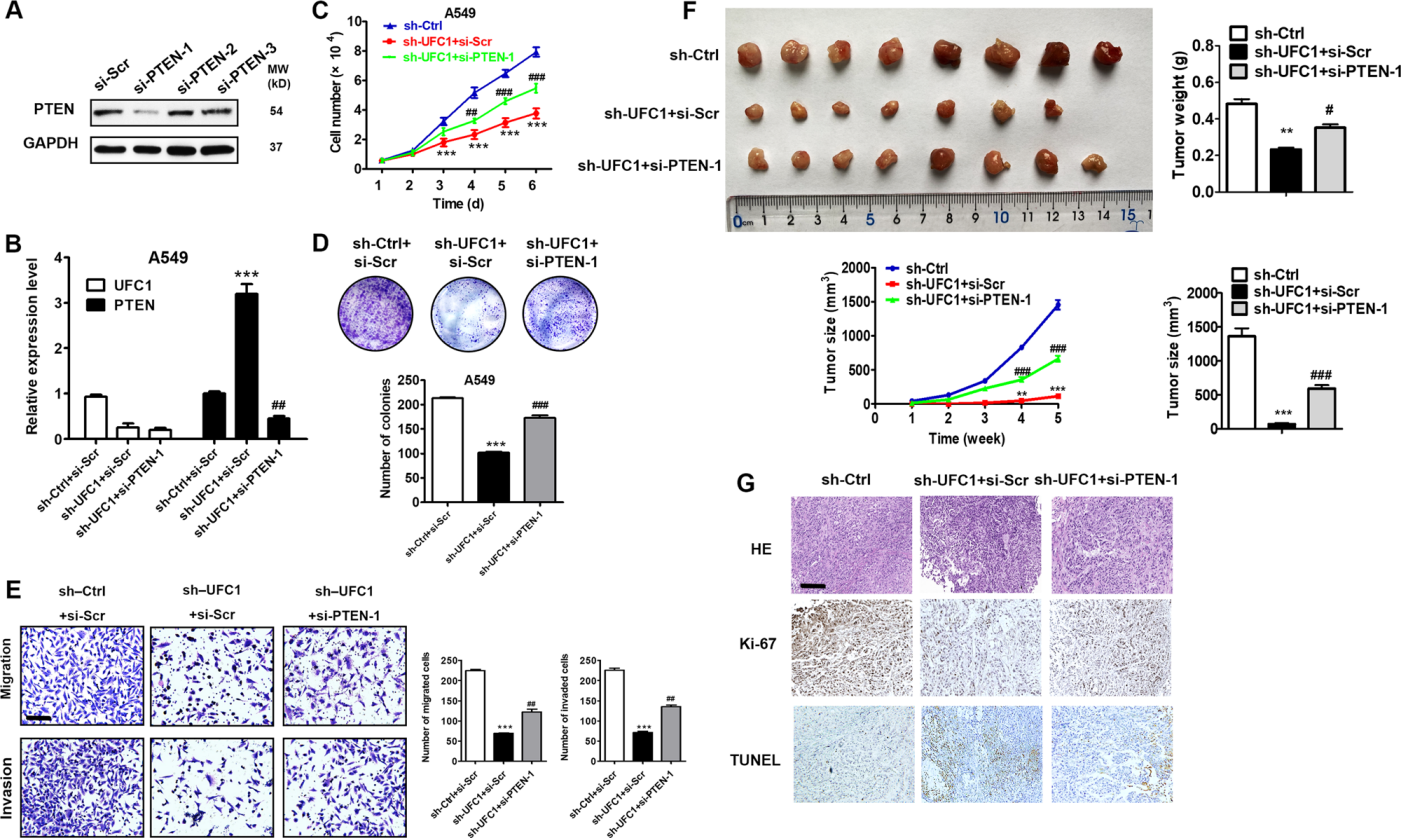
UFC1 promotes NSCLC progression via the regulation of PTEN. **a** QRT-PCR analysis of PTEN knockdown efficiency in A549 cells. **b** QRT-PCR analysis of UFC1 and PTEN expression in A549 cells transfected with sh-UFC1 alone or sh-UFC1+si-PTEN. **c** Cell counting assays for A549 cells transfected with sh-UFC1 alone or sh-UFC1+si-PTEN. **d** Colony formation assays for A549 cells transfected with sh-UFC1 alone or sh-UFC1+si-PTEN. **e** Transwell migration and matrigel invasion assays for A549 cells transfected with sh-UFC1 alone or sh-UFC1+ si-PTEN. **f** Tumor growth curves, tumor weights and sizes of mice injected with A549 cells transfected with sh-UFC1 alone or sh-UFC1+si-PTEN. **g** HE, Ki-67, and TUNEL staining in tumor tissues from mice injected with A549 cells transfected with sh-UFC1 alone or sh-UFC1+si-PTEN. The experiments were repeated for three times. Scale bar: 100 μm. *P < 0.05, **P < 0.01, ***P < 0.001, compared to sh-Ctrl+si-Scr group; ^#^*P* < 0.05, ^##^*P* < 0.01, ^###^*P* < 0.001, compared to sh-Ctrl+si-Scr group.

We then conducted rescue experiments to evaluate the importance of PTEN regulation in UFC1’s promoting role in NSCLC progression. PTEN siRNA was co-transfected with UFC1 shRNA into A549 cells. The results of qRT-PCR indicated that the upregulation of PTEN by UFC1 knockdown was reversed by PTEN siRNA (Fig. 5a, b). The results of cell counting and colony formation assays demonstrated that the proliferation of A549 cells co-transfected with sh-UFC1 and si-PTEN was increased compared to that of A549 cells transfected with sh-UFC1 alone (Fig. 5c, d). PTEN siRNA co-transfection also reversed the inhibiting effects of UFC1 knockdown on cell migration and invasion in A549 cells (Fig. 5e). Moreover, A549 cells that were transfected with sh-Ctrl, sh-UFC1 or sh-UFC1 + si-PTEN were injected into nude mice subcutaneously. The size and weight of tumors in sh-UFC1 group were obviously smaller than that in sh-Ctrl group at the end of experiments (Fig. 5f). However, the size and weight of tumors in sh-UFC1 + si-PTEN group were higher than that in sh-UFC1 alone group (Fig. 5f). The results of immunohistochemical analysis showed that tumor tissues in sh-UFC1 group had more TUNEL-positive and less Ki-67 cells than that in sh-Ctrl group. On the contrary, tumor tissues in sh-UFC1 + si-PTEN group had less TUNEL-positive and more Ki-67 positive cells than that in sh-UFC1 alone group (Fig. 5g). Collectively, these results suggest that UFC1 exhibits oncogenic roles in NSCLC partially through repression of PTEN expression.

### NSCLC cells derived exosomes deliver UFC1 to promote cancer progression by downregulating PTEN

**Fig. 6:**
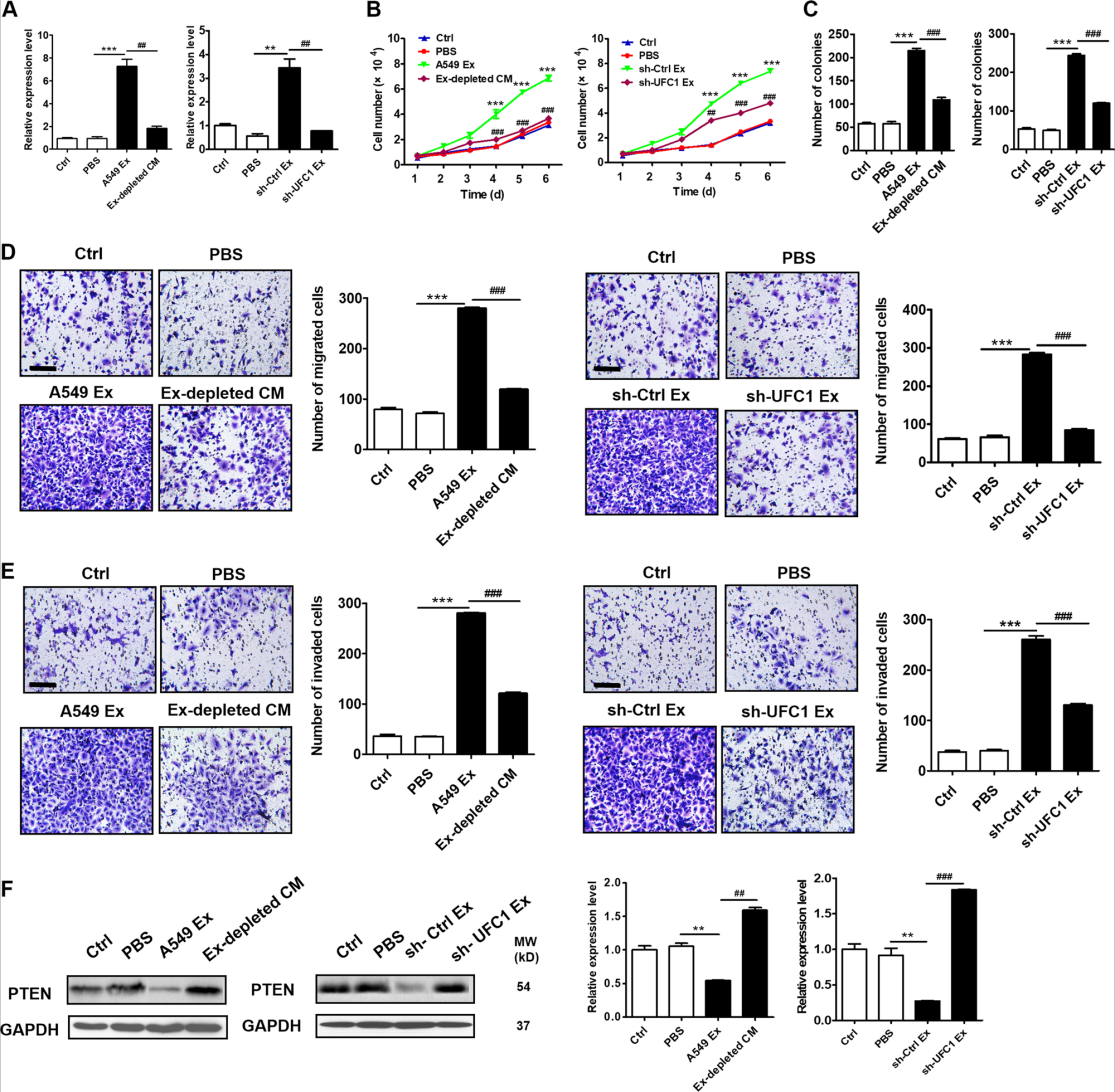
NSCLC cells derived exosomes deliver UFC1 to promote NSCLC progression by downregulating PTEN. **a** QRT-PCR analysis of UFC1 expression in A549 cells treated with PBS, exosomes-depleted conditioned medium (Ex-depleted CM), exosomes from sh-Ctrl and sh-UFC1 transfected A549 cells. **b**–**e** Cell counting (**b**), colony formation (**c**), transwell migration (**d**), and matrigel invasion (**e**) assays for A549 cells treated with PBS, Ex-depleted CM, or exosomes from sh-Ctrl and sh-UFC1 transfected A549 cells. **f** QRT-PCR and western blot analysis of PTEN expression in A549 cells treated with PBS, Ex-depleted CM, or exosomes from sh-Ctrl and sh-UFC1 transfected A549 cells. The experiments were repeated for three times. Scale bar: 100 μm. *P < 0.05, **P < 0.01, ***P < 0.001, compared to *P*BS group; ^#^*P* < 0.05, ^##^*P* < 0.01, ^###^*P* < 0.001, compared to A549 Ex group; ^#^*P* < 0.05, ^##^*P* < 0.01, ^###^*P* < 0.001, compared to sh-Ctrl Ex group.

The presence of UFC1 in serum exosomes of NSCLC patients led us to hypothesize that UFC1 may be delivered in the circulation in an exosome form. We then incubated A549 cells with exosomes or exosomes-depleted conditioned medium (Ex-depleted CM) from A549 cells. The results of qRT-PCR showed that the incubation with exosomes but not Ex-depleted CM led to an increase in UFC1 expression in A549 cells (Fig. 6a). We further extracted exosomes from A549 cells transfected with sh-Ctrl or sh-UFC1 and incubated these exosomes with A549 cells for 24 h. The results of qRT-PCR showed that the incubation with exosomes from sh-Ctrl cells led to an increase in UFC1 expression level in A549 cells (Fig. 6a). However, the incubation with exosomes from sh-UFC1 cells had minimal effect. We then wanted to know the effect of exosome-mediated delivery of UFC1 on NSCLC progression. Exosomes and Ex-depleted CM were applied to A549 cells for cell counting, colony formation, migration and invasion assays. The results showed that exosomes could promote the proliferation (Fig. 6b, c), migration (Fig. 6d) and invasion (Fig. 6e) of A549 cells while Ex-depleted CM had no apparent effect. In addition, compared to exosomes from sh-Ctrl cells, exosomes from sh-UFC1 cells had lower effect to promote the proliferation, migration and invasion of A549 cells (Fig. 6b–e). Furthermore, exosomes from sh-Ctrl cells could inhibit PTEN expression in A549 cells while exosome-depleted CM and exosomes from sh-UFC1 cells had no such effects (Fig. 6f). In summary, these data suggest that NSCLC cells derived exosomes may transmit UFC1 to promote cancer progression by downregulating PTEN.

## Discussion

LncRNAs have been considered as promising diagnostic and prognostic markers for various cancers including NSCLC. The previous studies have revealed various lncRNAs as important players in NSCLC progression. In this study, we analyzed the expression of lncRNA UFC1 in NSCLC patients and investigated its biological roles in NSCLC progression. Our results showed that UFC1 expression was significantly upregulated in NSCLC tissues compared to adjacent normal lung tissues, which is consistent with previous studies showing that UFC1 is upregulated in HCC^13^, colorectal cancer^14^, and gastric cancer^16^. We reported that UFC1 was highly expressed in serum and serum exosomes of NSCLC patients compared to that of pneumonia patients and healthy controls, indicating that UFC1 may represent a convenient and reliable biomarker for the diagnosis of NSCLC patients. Due to the small cohort of samples used in this study, the clinical significance of UFC1 (including serum UFC1 and exosomal UFC1) still needs to be further verified in a larger cohort of samples. The value of UFC1 in the prognosis of NSCLC patients also needs to be investigated in future studies. Moreover, the results of gain- and loss-of-function studies showed that UFC1 overexpression promoted while UFC1 knockdown suppressed the proliferation, migration and invasion of NSCLC cells in vitro and inhibited the tumorigenesis of NSCLC cells in vivo, indicating that UFC1 plays oncogenic roles in NSCLC progression.

Increasing evidence suggest that lncRNAs regulate the expression of downstream genes by acting as competing endogenous RNAs (ceRNAs) and binding to RNA-binding proteins (RBPs). LINC00673 promotes NSCLC cell proliferation, migration, and invasion by sponging miR-150-5p^17^. UCA1 upregulates ERBB4 expression through competitively sponging miR-193a-3p, thus enhancing the proliferation of NSCLC cells^18^. AFAP1-AS1 promotes NSCLC cell migration and invasion through positive regulation of AFAP1 protein expression^19^. In LKB1-inactivated NSCLC cells, LINC00473 interacts with NONO, a component of the cAMP signaling pathway to facilitate CRTC/CREB-mediated transcription. LINC00963 is highly expressed in human NSCLC and correlates with poor prognosis. LINC00963 promotes NSCLC metastasis by interacting with the glycolytic kinase PGK1 and preventing its ubiquitination, leading to activation of the oncogenic AKT/mTOR signaling pathway^20^. As estimated, over 20% of lncRNAs could bind to the components of the Polycomb Repressive Complex 2 (PRC2) to regulate target gene expression^21^. EZH2 is a histone methyltransferase and the core functional enzymatic component of PRC2. The recent studies have shown that EZH2 regulates diverse cellular processes and participates in cancer development, progression and metastasis. Many lncRNAs bind to EZH2 to epigenetically silence gene expression in NSCLC cells^22–34^, such as TUG1^22^, ANRIL^23^, PANDAR^24^, PVT1^25^, and PCAT6^26^. These lncRNAs interact with EZH2 and regulate the expression of distinct target genes, including p21, KLF2, p57, Bcl2, LATS2, and p57. In this study, we revealed that UFC1 overexpression decreased while UFC1 knockdown increased PTEN expression in NSCLC cells. UFC1 repressed PTEN expression via the recruitment of EZH2 and the subsequent epigenetic modification. Our results are consistent with those reported by Gan et al. showing that EZH2 induces EMT and pluripotency of gastric cancer cells by suppressing PTEN expression^34^.

PTEN regulates various cellular signaling pathways and plays important roles in cell survival, proliferation, and apoptosis^35^. Abnormal PTEN expression is associated with the pathogenesis of multiple diseases including cancer. Recently, noncoding RNAs have been reported to regulate PTEN protein expression epigenetically or post-transcriptionally. Wang et al. show that Linc02023 specifically binds to PTEN and blocks its interaction with and ubiquitination by WWP2. Linc02023 regulates PTEN stability and suppresses tumorigenesis of colorectal cancer in a PTEN-dependent pathway^36^. LncRNA-BGL3 functions as a ceRNA for miR-17/93 cluster to regulate PTEN expression in leukemic cells. LncRNA-BGL3 overexpression induces apoptosis of leukemic cells through the upregulation of PTEN and the inactivation of Akt pathway^37^. Herein, we reported another lncRNA UFC1 that could regulate PTEN expression at the epigenetic level, which adds more information for the regulatory network of PTEN in cancer. Although this study provides mechanistic insight into the function of UFC1 in NSCLC progression, it could not be excluded that UFC1 may exert its role in NSCLC through other mechanisms. We have previously shown that UFC1 promotes gastric cancer progression by acting as a ceRNA for miR-498. By using bioinformatic analysis (miRcode, miRDB, and Targetscan databases), we predicted that UFC1 might bind to miR-4470 and miR-338-3p, which are also regulating miRNAs for PTEN. Whether UFC1 can regulate PTEN by acting as a miRNA sponge for miR-4470 and miR-338-3p still needs further study and is not within the scope of this study.

Exosomes are now recognized as a new mode of communication and information exchange between cells. Exosomes secreted by tumor cells and stromal cells have been shown to participate in tumor initiation, growth, progression, metastasis, and drug resistance^38^. Exosomes contain specific repertoires of non-coding RNAs including lncRNAs, which may influence the function of recipient cells^39–41^. Exosome from sunitinib-resistant renal cancer cells could transfer lncARSR (lncRNA activated in RCC with sunitinib resistance) to sensitive cells, in which LncARSR competes with miR-34 and miR-449, promotes AXL and c-MET expression, reactivates RTKs, and mediates drug resistance^42^. Exosomal Sox2ot could upregulate Sox2 expression to promote pancreatic adenocarcinoma cancer (PDAC) cell invasion and metastasis by competitively binding to miR-200 family^43^. Exosomes from hypoxic bladder cancer cells express an increased level of lncRNA-UCA1 and could promote tumor growth and progression though EMT^44^. H19 is enriched in carcinoma- associated fibroblasts (CAFs)-derived exosomes, which in turn promotes the stemness and chemoresistance of CRC cells by activating the β-catenin pathway via acting as a ceRNA sponge for miR-141^45^. Moreover, exosomes are readily accessible in many types of human biofluids including blood, saliva, urine, and ascites, providing a useful maker for cancer diagnosis and prognosis. The plasma exosomal Sox2ot expression is high in PDAC patients and correlated with TNM stage and overall survival^43^. LncUEGC1 is confirmed to be remarkably upregulated in plasma exosomes of early stage gastric cancer (EGC) patients and has better diagnostic accuracy to discriminate EGC patients from healthy individuals and those with premalignant chronic atrophic gastritis than carcinoembryonic antigen (CEA)^46^. We showed that UFC1 was highly expressed in exosomes from NSCLC cells and serum of NSCLC patients. Exosomal UFC1 could promote NSCLC cell proliferation, migration and invasion, indicating the multifaceted roles of exosomal lncRNAs in NSCLC. Lin and his colleagues demonstrate that normal cells secrete exosomal PTENP1 and transmit it to bladder cancer (BC) cells to inhibit their malignant behaviors through the upregulation of PTEN by competitively binding to miRNA-17^47^. We reported that NSCLC cells derived exosomal UFC1 inhibited PTEN expression to promote NSCLC progression by EZH2-mediated epigenetic silencing, suggesting that PTEN is regulated by exosomal lncRNAs via distinct mechanisms. In the future study, we will further investigate the clinical significance of circulating UFC1 in NSCLC patients and clarify the specific mechanism for the roles of UFC1 in NSCLC.

## Conclusion

**Fig. 7:**
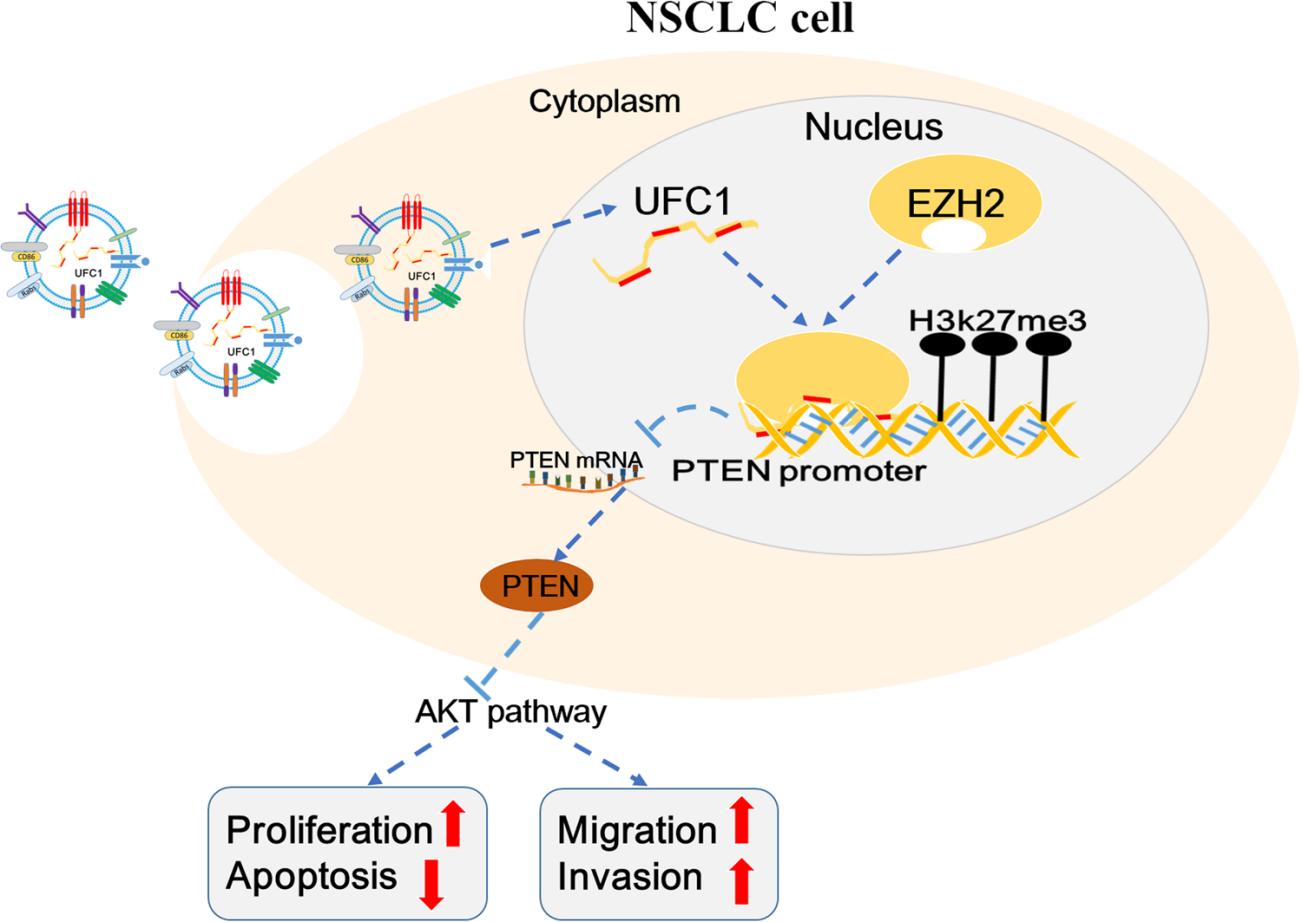
Proposed model for the oncogenic roles of UFC1 in NSCLC. Exosome-transmitted UFC1 epigenetically silences PTEN expression via binding to EZH2, which promotes Akt pathway activation and increases cell proliferation, migration and invasion in NSCLC.

In conclusion, our present study showed that UFC1 was upregulated in NSCLC cells and tumor tissues, serum and serum exosomes of NSCLC patients. Exosomal UFC1 could be a promising biomarker for the diagnosis of NSCLC. Knockdown of UFC1 significantly suppressed NSCLC cell proliferation, migration and invasion and tumorigenesis while induced cell apoptosis. UFC1 exerted oncogenic roles in NSCLC partly through epigenetically repressing PTEN expression via its binding to EZH2 (Fig. 7). These data indicate that UFC1 may function as an oncogene and exosome-transmitted UFC1 may play a significant role in NSCLC progression.

## Supplementary information

Supplementary figure legends

FigureS1

FigureS2

Supplementary tables

## Data Availability

All of the data and material in this paper are available when requested.
