# Supplementary material for: Exosome-transmitted lncRNA UFC1 promotes non-small-cell lung cancer progression by EZH2-mediated epigenetic silencing of PTEN expression
Source: Cell Death Dis. 2020 Apr 2;11(4):215. doi: 10.1038/s41419-020-2409-0 (PMC7118073; doi:10.1038/s41419-020-2409-0)
Supplement: Supplementary file 1 — Supplementary figure legends [file 41419_2020_2409_MOESM1_ESM.docx]

**Figure S1 UFC1 knockdown inhibits proliferation, migration and invasion of NSCLC cells.** (A) QRT-PCR analysis of UFC1 knockdown efficiency A549 cells. (B) Cell counting assays for sh-Ctrl and sh-UFC1 transfected A549 cells. (C) Colony formation assays for sh-Ctrl and sh-UFC1 transfected A549 cells. (D-E) Transwell migration (D) and matrigel invasion (E) assays for sh-Ctrl and sh-UFC1 transfected A549 cells. The experiments were repeated three times. Scale bar: 100 μm. **P*<0.05, ***P*< 0.01, ****P*<0.001, compared to sh-Ctrl group.

**Figure S2 EZH2 knockdown inhibits proliferation, migration and invasion of NSCLC cells.** (A) QRT-PCR analysis of EZH2 knockdown efficiency A549 cells. (B) Cell counting assays for sh-Ctrl and sh-EZH2 transfected A549 cells. (C) Colony formation assays for sh-Ctrl and sh-EZH2 transfected A549 cells. (D-E) Transwell migration (D) and matrigel invasion (E) assays for sh-Ctrl and sh-EZH2 transfected A549 cells. The experiments were repeated three times. Scale bar: 100 μm. **P*<0.05, ***P*< 0.01, ****P*<0.001, compared to si-Scr group.
