# Supplementary figures and images for: Exosome-transmitted lncRNA UFC1 promotes non-small-cell lung cancer progression by EZH2-mediated epigenetic silencing of PTEN expression
Source: Cell Death Dis. 2020 Apr 2;11(4):215. doi: 10.1038/s41419-020-2409-0 (PMC7118073; doi:10.1038/s41419-020-2409-0)

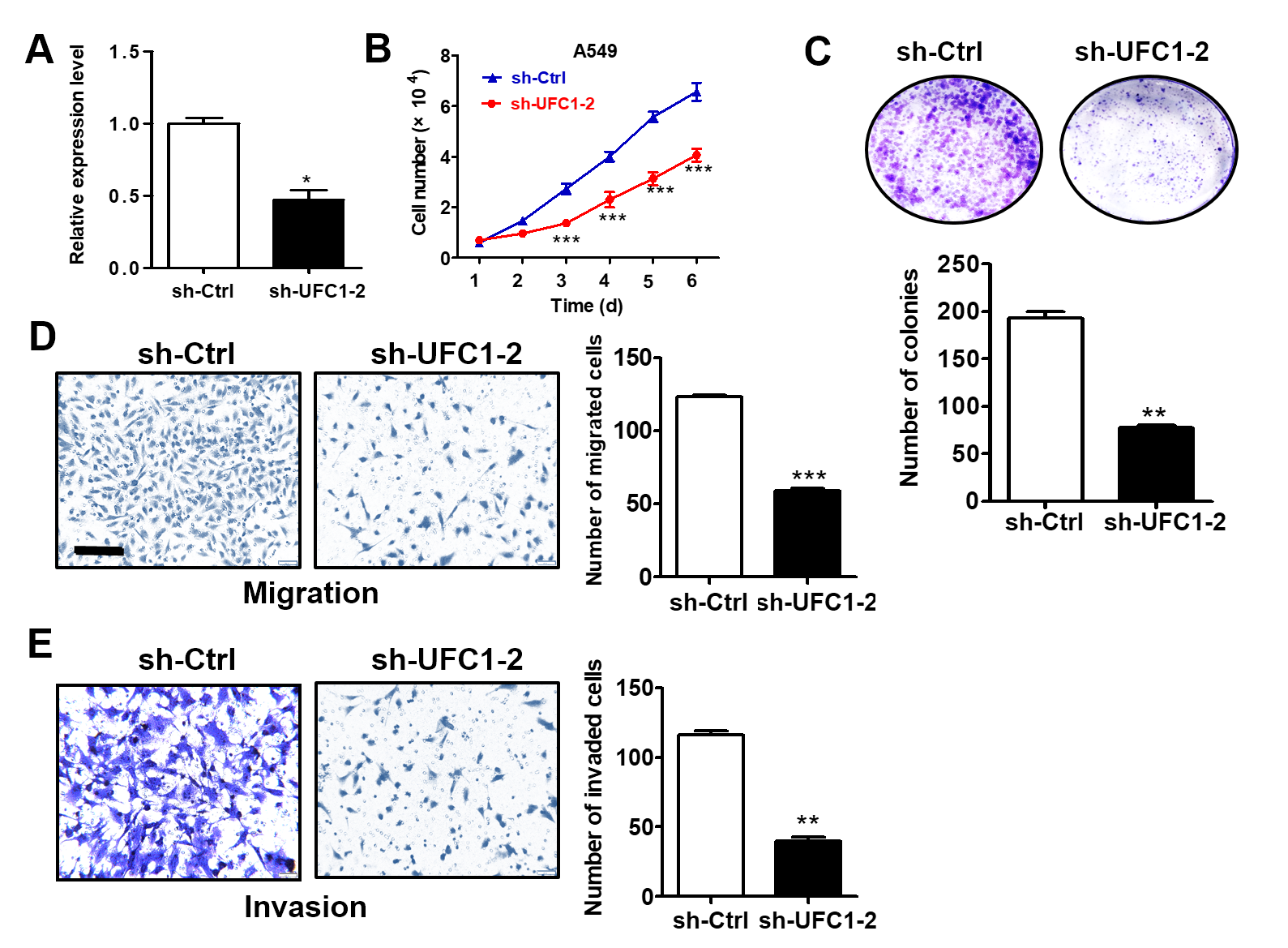

Supplement: Supplementary file 2 — FigureS1 [file 41419_2020_2409_MOESM2_ESM.tif]

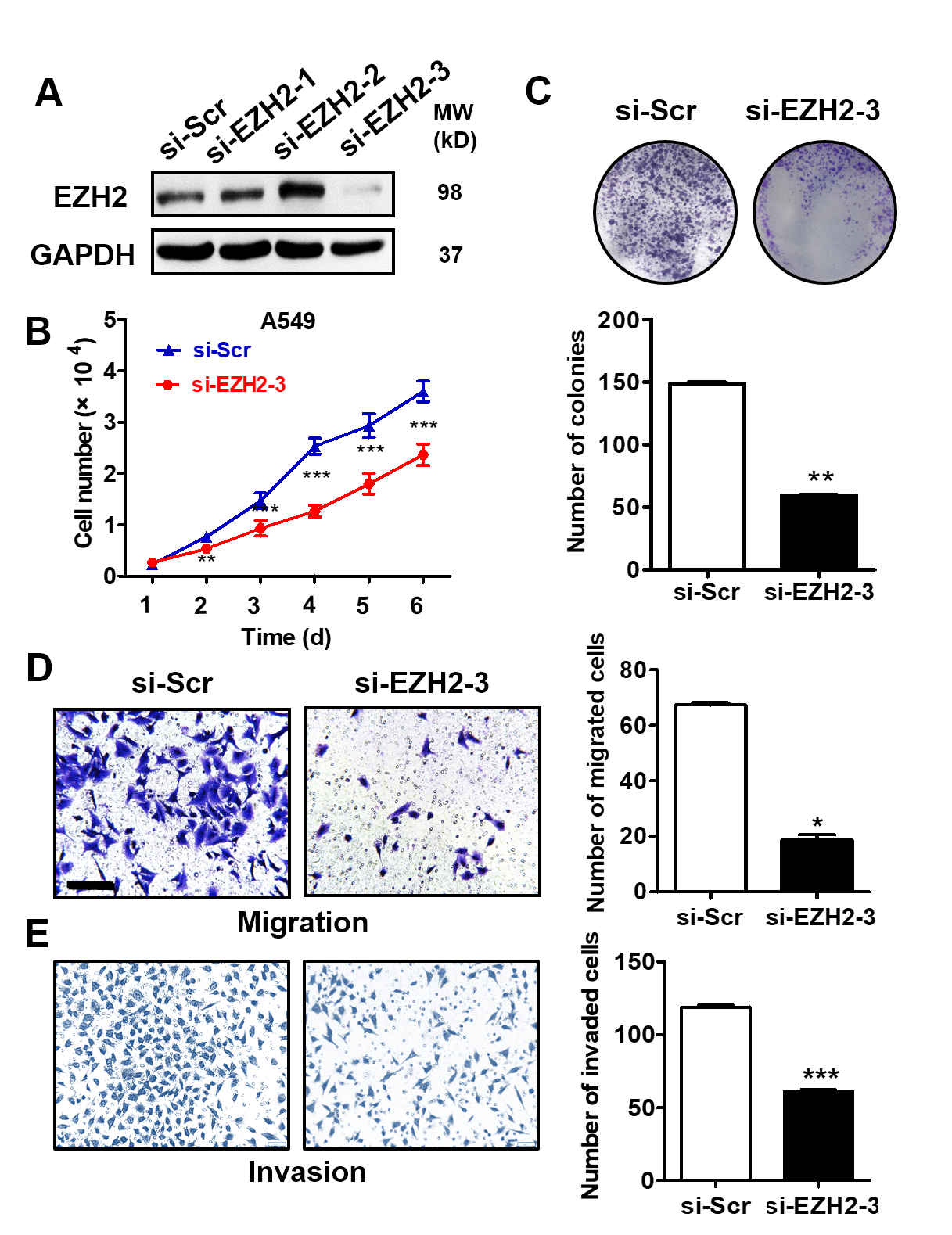

Supplement: Supplementary file 3 — FigureS2 [file 41419_2020_2409_MOESM3_ESM.tif]
