# Supplementary material for: Exosome-transmitted lncRNA UFC1 promotes non-small-cell lung cancer progression by EZH2-mediated epigenetic silencing of PTEN expression
Source: Cell Death Dis. 2020 Apr 2;11(4):215. doi: 10.1038/s41419-020-2409-0 (PMC7118073; doi:10.1038/s41419-020-2409-0)
Supplement: Supplementary file 4 — Supplementary tables [file 41419_2020_2409_MOESM4_ESM.docx]

**Table S1. Primer sequences of target genes**

| Target | Sequence (5’-3’) | Size (bp) | Tm (^o^C) |
| --- | --- | --- | --- |
| U6 | F:5’-CTCGCTTCGGCAGCACA-3’ | 94 | 55 |
|  | R:5’-AACGCTTCACGAATTTGCGT-3’ |  |  |
| UFC1 | F:5'-TCCAACCTGAGTGACATAGCGA-3' | 172 | 55 |
|  | R:5’-CTGACCTCCAACTCCAACGAAT-3’ |  |  |
| E-cadherin | F:5’-CGCATTGCCACATACACTCT-3’ | 252 | 55 |
|  | R:5’-TTGGCTGAGGATGGTGTAAG-3’ |  |  |
| N-cadherin | F:5’-AGTCAACTGCAACCGTGTCT-3’ | 337 | 55 |
|  | R:5’-AGCGTTCCTGTTCCACTCAT-3’ |  |  |
| Slug | F:5’-CCTGGTTGCTTCAAGGACAC-3’ | 395 | 55 |
|  | R:5’-TCCATGCTCTTGCAGCTCTC-3’ |  |  |
| Snail | F:5’-GCGAGCTGCAGGACTCTAAT-3’ | 310 | 55 |
|  | R:5’-GCCTCCAAGGAAGAGACTGA-3’ |  |  |
| Twist | F:5’-ACGAGCTGGACTCCAAGATG-3’ | 484 | 55 |
|  | R:5’-GGCACGACCTCTTGAGAATG-3’ |  |  |
| ZEB1 | F:5’-CAGAAGCCAGTGGTCATGAT-3’ | 247 | 55 |
|  | R:5’-GACTGCGTCACATGTCTTTG-3’ |  |  |
| Bax | F:5’-CACCAGCTCTGAGCAGATCAT-3’ | 214 | 55 |
|  | R:5’-GATCAGTTCCGGCACCTTG-3’ |  |  |
| Bcl-2 | F:5’-GGATCCAGGATAACGGAGGC-3’ | 150 | 55 |
|  | R: 5'-CCAGATAGGCACCCAGGGT-3' |  |  |
| Cyclin D1 | F: 5'-CCGAGAAGCTGTGCATCTAC-3' | 221 | 55 |
|  | R: 5'-CTTCACATCTGTGGCACAGAG-3' |  |  |
| PTEN | F: 5'-CGACGGGAAGACAAGTTCAT-3' | 163 | 58 |
|  | R: 5'-AGGTTTCCTCTGGTCCTGGT-3' |  |  |

**Table S2. The sequences of shRNAs and siRNAs**

| Target | Sequence (5’-3’) |
| --- | --- |
| sh- control | GATCCGGAGCTCATGGGTCC TTTGTATCGGTACCGATACA  AAGGACCCATGAGCTTTTTTG |
| sh- UFC1-1 | CCGGAAGCACAGTGGTCTAAAAGTACTCGAGTACTT  TTAGACCACTGTGCTTTTTTTG |
| sh- UFC1-2 | CCGGCTGTAGAAGGTTGAAGGGAAACTCGAGTTT  CCCTTCAACCTTCTACAGTTTTTG |
| si-control | UUCUCCGAACGUGUCACGUTT |
| si- EZH2 | GAGGGAAAGUGUAUGAUAATT |
| si- PTEN | CCACCACAGCUAGAACUUATT |

**Table S3. The sequences of ChIP primers**

| Target | Sequence (5’-3’) | Size (bp) | Tm (^o^C) |
| --- | --- | --- | --- |
| PTEN | F: 5'-GGAGGCAGCCGTTCGGAGGATTATT-3' | 201 | 58 |
|  | R: 5'-GGAAATGGCTCTGGACTTGGCGGTA-3' |  |  |

**Table S4. The association between lncUFC1 expression levels (–ΔΔCt) in tumor tissues and the clinicopathological features of lung cancer patients**

| Features | Number | UFC1 | | *P* value |
| --- | --- | --- | --- | --- |
|  |  | High | Low |  |
| Gender |  |  |  | 0.577 |
| Male | 46 | 26 | 20 |  |
| Female | 22 | 14 | 8 |  |
| Age (year) |  |  |  |  |
| <65 | 33 | 15 | 18 | 0.030 |
| ≥65 | 35 | 25 | 10 |  |
| TNM |  |  |  |  |
| Ⅰ-Ⅱ | 30 | 17 | 13 | 0.486 |
| Ⅲ-IV | 29 | 19 | 10 |  |
| Lymphatic metastasis |  |  |  |  |
| Absent | 27 | 15 | 23 | 0.487 |
| Present | 31 | 20 | 11 |  |
| Tumor infiltration |  |  |  |  |
| Absent | 40 | 20 | 20 | 0.021 |
| Present | 18 | 15 | 3 |  |
| Vascular invasion |  |  |  |  |
| Absent | 57 | 34 | 23 | 1.000 |
| Present | 1 | 1 | 0 |  |
| Tumor size (cm) |  |  |  | 0.298 |
| <5 | 40 | 22 | 18 |  |
| ≥5 | 25 | 17 | 8 |  |
| Tumor location |  |  |  |  |
| Left lung | 28 | 16 | 12 | 0.973 |
| Right lung | 33 | 19 | 14 |  |
